# Manipulation of the *Tyrosinase* gene permits improved CRISPR/Cas editing and neural imaging in cichlid fish

**DOI:** 10.1038/s41598-021-94577-8

**Published:** 2021-07-23

**Authors:** Cheng-Yu Li, Joshua R. Steighner, Garrett Sweatt, Tod R. Thiele, Scott A. Juntti

**Affiliations:** 1 Department of Biology, University of Maryland, College Park, MD USA; 2 Department of Biological Sciences, University of Toronto, Scarborough, ON Canada

**Keywords:** Non-model organisms, CRISPR-Cas9 genome editing, Evolutionary biology, Development, Ichthyology

## Abstract

Direct tests of gene function have historically been performed in a limited number of model organisms. The CRISPR/Cas system is species-agnostic, offering the ability to manipulate genes in a range of models, enabling insights into evolution, development, and physiology. *Astatotilapia burtoni*, a cichlid fish from the rivers and shoreline around Lake Tanganyika, has been extensively studied in the laboratory to understand evolution and the neural control of behavior. Here we develop protocols for the creation of CRISPR-edited cichlids and create a broadly useful mutant line. By manipulating the *Tyrosinase* gene, which is necessary for eumelanin pigment production, we describe a fast and reliable approach to quantify and optimize gene editing efficiency. *Tyrosinase* mutants also remove a major obstruction to imaging, enabling visualization of subdermal structures and fluorophores in situ. These protocols will facilitate broad application of CRISPR/Cas9 to studies of cichlids as well as other non-traditional model aquatic species.

## Introduction

Cichlid fishes live in a wide variety of ecological, sensory, and social environments and have evolved elaborate variations in physiology and behavior^1,2^. Levels of behavioral and morphological diversity are extraordinarily high in African cichlids as a result of an explosive and ongoing radiation^3^, providing a valuable opportunity to link genetic mechanisms to a wide variety of phenotypes. Numerous genes have been hypothesized to regulate physiological processes, development, or behavior as implicated through gene expression or pharmacology. Genetic tools have been developed for mapping the control of traits to genomic loci, including whole-genome sequences for dozens of cichlid species^3–11^. While these sequences facilitate candidate gene discovery, a formal demonstration of gene function requires the experimental manipulation of individual genes.

We use the mouthbrooding African cichlid, *Astatotilapia burtoni*, from Lake Tanganyika as a model due to key advantages over other cichlids. *A. burtoni* is similar to the common ancestors of the prodigious *Haplochromine* cichlid radiation in Lakes Tanganyika, Victoria and Malawi^12,13^. This species is therefore a good candidate for testing the functions of many genes implicated in other *Haplochromine* species. Further, husbandry in *A. burtoni* is straightforward: they breed readily in the lab and female mouthbrooding of embryos allows for easy collection of eggs for use in gene editing. *A. burtoni* have a ~ 4 month generation time, and large females produce ~ 100 eggs per month. Their mating behavior does not have a strong seasonal component, allowing for year-round gene editing in the laboratory. The *A. burtoni* genome is well-annotated^4^, enabling the determination of genetically homologous loci. Lastly, a large body of literature exists using *A. burtoni*, particularly behavioral and neurobiological studies^14^.

Until recently, direct tests of gene function through reverse genetics were feasible in only a limited number of species due to the difficulty of manipulating embryos and the low efficiency of gene editing tools. CRISPR/Cas is an RNA-mediated adaptive immune system found in bacteria that protects against the invasion of viruses and plasmids^15^. Its adaptation for use in eukaryotic genomes^16^ has revolutionized reverse genetic approaches in a wide variety of species, enabling targeted modification of genomes without a reliance on homologous recombination or expensive reagents^17^. In brief, the system makes use of an endonuclease (Cas9) which complexes with a tracrRNA and a guide RNA (gRNA) that determines the site of genome targeting. The gRNA binds the target locus via Watson–Crick base pairing, and Cas9 creates a double-stranded break in DNA. Cellular DNA repair machinery utilizes either non-homologous end joining (NHEJ) or homology-directed repair (HDR) to repair the chromosome. NHEJ is an imprecise process that often leads to insertions or deletions (indels). If an indel results in a frameshift mutation of the coding sequence of a gene, a loss-of-function mutation is likely to result. HDR utilizes a DNA template to guide repair, offering the ability to insert exogenous DNA sequences. In practice, however, template insertion via HDR is difficult to achieve in fish species, though some progress has been made^18–22^. We focus on the use of CRISPR/Cas to generate loss-of-function (LoF) mutations, as they can be rapidly and inexpensively created, and these mutations provide insights into development, physiology, behavior, and evolution.

In this paper we generate LoF mutations in the *Tyrosinase* (*Tyr1*) gene, which encodes a key enzyme for eumelanin synthesis. This strategy has been useful in zebrafish, medaka, and lizard mutagenesis^23–25^. The mutant phenotype of nonpigmented melanocytes is easily visible early in development and stable in adults, making *Tyr1* gRNA microinjections an efficient tool to assess mutagenesis rates and troubleshoot CRISPR/Cas9 protocols. Further, these eumelanin-deficient mutants enable analysis of visual signaling and facilitate in vivo activity-based imaging of both embryonic and adult brains. Here we outline the processes that we have developed to generate mutant and transgenic cichlids, including genetic target selection, reagent preparation, embryo manipulation, genotyping, and husbandry. These approaches should be applicable to species beyond *A. burtoni*, including cichlids more generally and other fish species that can be raised in aquatic laboratories.

**Figure 1:**
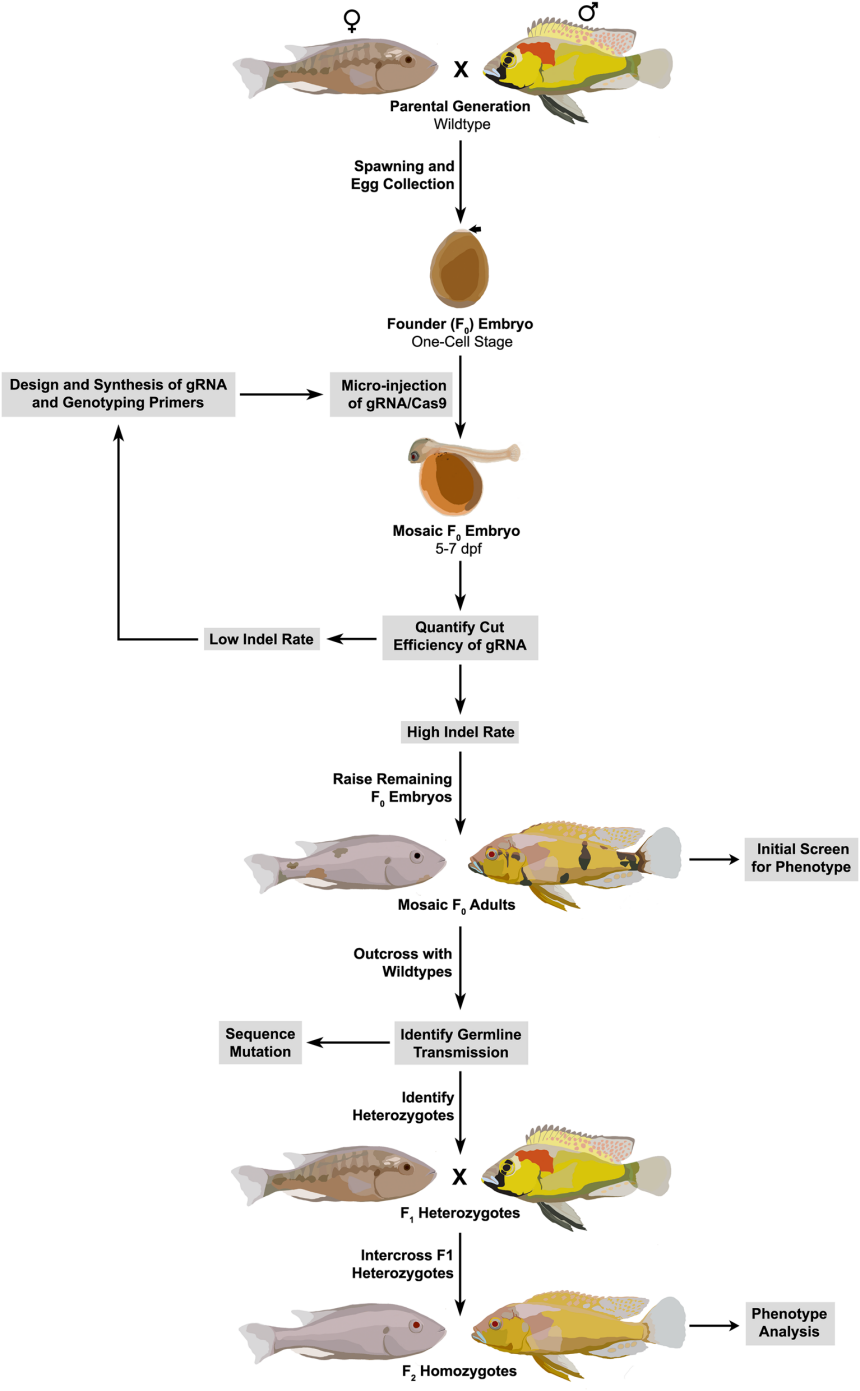
Overview of CRISPR/Cas9 mutagenesis workflow**.** Single-cell wildtype embryos are collected for microinjection with gRNA and Cas9 protein. At 5–7 days post-fertilization (dpf), indel mutation efficiency is assessed via PCR fragment size analysis. If indel rates are low, troubleshooting is initiated. Targeting the *Tyrosinase* (*Tyr1*) locus facilitates this process due to its visible phenotype. New gRNAs may improve mutagenesis rates. If indel rates are high, adult injected (F_0_) fish are screened for initial phenotypes and outcrossed to wildtype animals. Resulting F_1_ animals are screened for heterozygotes and sequenced to determine the mutation. Adult F_1_ may be intercrossed, and F_2_ offspring are used to determine phenotypes in homozygous mutants. Routine outcrosses are recommended to mitigate the impact of off-target effects.

The general approach to gene editing in cichlids is described here, using a targeting strategy for a pigmentation gene, *Tyrosinase* (Fig. 1). First, we collect and inject single-cell embryos with CRISPR gRNA and Cas9 to induce DNA breaks and subsequent mutations in the gene of interest. Then we screen injected fish by PCR to identify animals carrying indels at the desired site and select genetic mosaic animals to breed. Offspring of injected animals are screened for mutations to identify heterozygous founders of mutant lines. We cross heterozygous animals to generate homozygous mutant cichlids (plus wildtype and heterozygous controls) that we assay for phenotypes of interest.

The success of gene editing depends on the efficiency of several steps. First, a large number of embryos should be obtained for injection because there is embryo death due to natural causes as well as the injection itself. Further, the gene target may not be mutated in the germline, which is necessary to establish a genetic line. Finally, not all mutations are likely to be of large effect, and thus it is desirable to recover frameshift mutations that are large and therefore facilitate easy genotyping. Here, we describe approaches to optimize each step and maximize the number of edited animals recovered. The genome editing workflow, summarized in Fig. 1, consists of the design and synthesis of gRNA and genotyping primers, spawning and egg collection, micro-injection of gRNA/Cas9, determining and quantifying cut efficiency of gRNA, and recovery and maintenance of mutant lines. While we focus on the generation of loss-of-function alleles through frameshift mutations, the approaches here can be generalized to the creation of other mutant lines. The detailed protocols provided in supplementary materials will improve gene editing efficiency for a variety of cichlid species.

## Results

### Optimizing cichlid embryo recovery and survival rates

**Figure 2:**
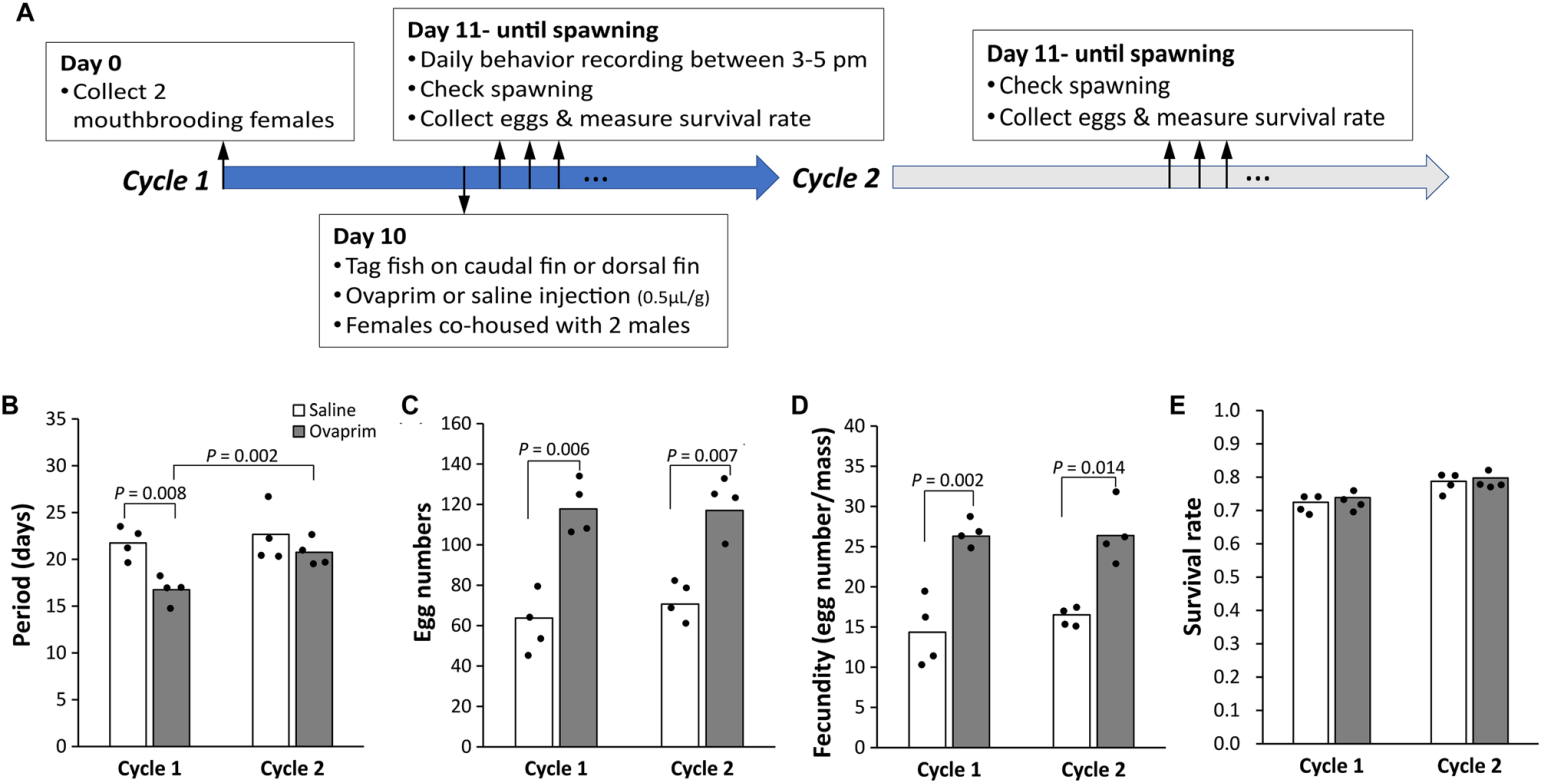
Ovaprim increases fecundity in *A. burtoni*. (**A**) Workflow for testing Ovaprim effects. We injected Ovaprim (0.5 μL per g of fish) into females 10 days after spawning and monitored for spawning behavior in the following two reproductive cycles. Females were monitored every day until they were observed carrying a brood. (**B**) Ovaprim advanced the reproductive cycle around 5 days, but timing of the following cycle was unchanged. (**C**) Ovaprim increased number of eggs laid compared with saline-injected females, an increase observed in the following reproductive cycle as well. (**D**) Ovaprim increased number of eggs laid, even when controlling for mass of female. (**E**) Ovaprim did not affect the overall survival rates of the embryos. Two-tailed Mann–Whitney *U* test was used to compare groups.

To improve gene editing, we first sought to maximize recovery of fertilized single-cell embryos. We asked whether spawning could be facilitated by intraperitoneal injections of Ovaprim (Syndel), a commercially available mixture of gonadotropin-releasing hormone analogue and dopamine receptor antagonist^26^. We found that Ovaprim injection (Fig. 2A) shortens the time to egg laying by ~ 5 days (Fig. 2B). It also increases the yield of eggs laid by ~ 2-fold (Fig. 2C,D), without affecting egg viability (Fig. 2E). Interestingly, the increased fecundity effect of Ovaprim is observed in the following ovarian cycle, though period of the cycle returns to normal.

**Figure 3:**
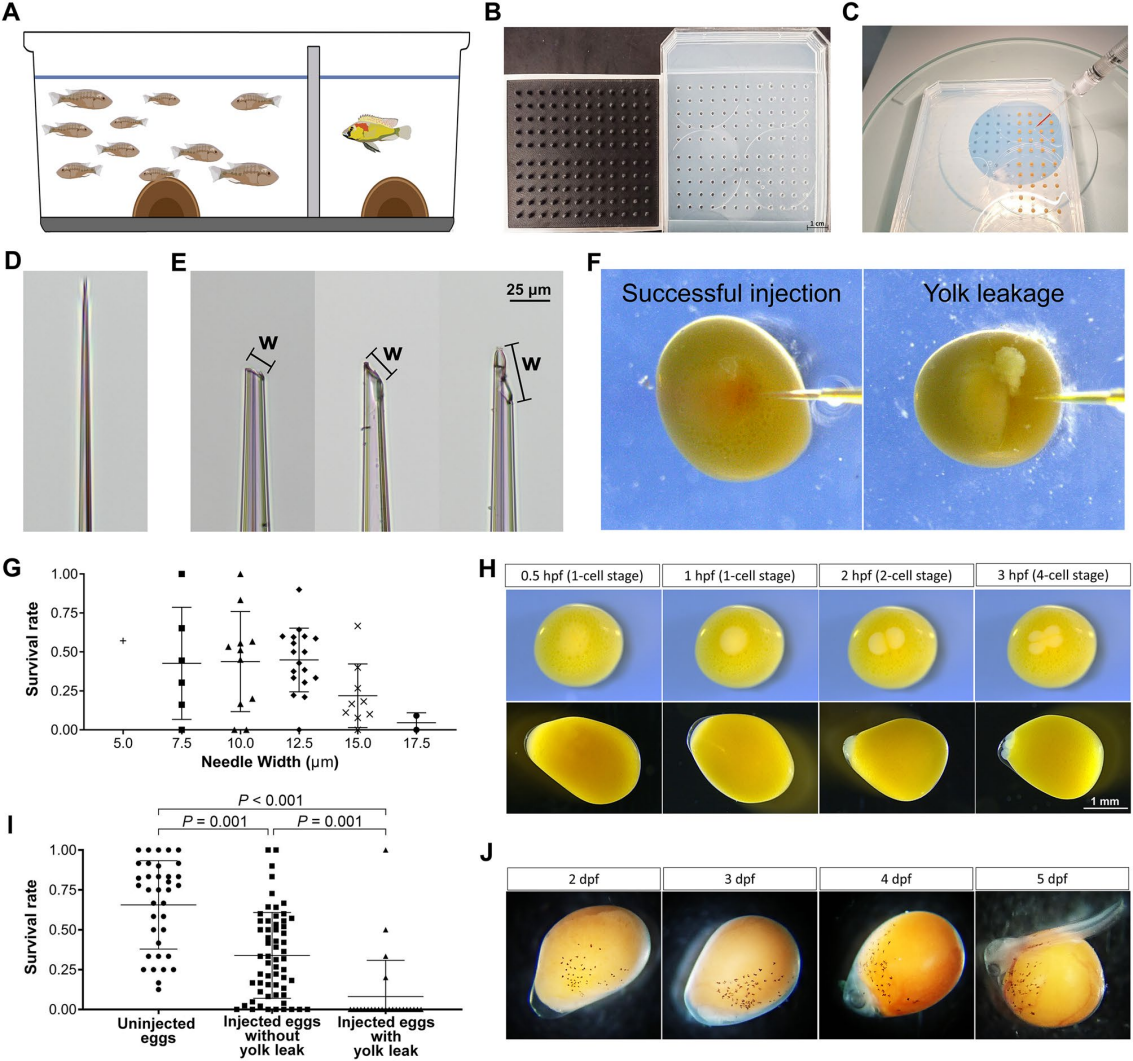
Cichlid embryo collection and injection. (**A**) 20–30 L breeding tanks contain ~ 10 adult females separated by a transparent barrier from a stud male. (**B**) A 3D-printed mold (left) for making gel embryo-holder (right). (**C**) Single-cell embryos loaded for injection with CRISPR components. (**D**,**E**) Example of injection needles before (**D**) and after (**E**) tip opening. Note that the variation in the shape of microinjection needles could impact both the volume of reagents injected and the extent of damage to the embryo. ‘W’ represents ‘needle width’, which is defined as the long axis of the needle’s angled opening. (**F**) An example of successful injection (left) versus yolk leakage (right). The survival rate of embryos at 5–7 dpf, dependent on (**G**) the outer diameter of the needle used and (**I**) the presence of yolk leakage. Each data point consists of a single brood injected by a unique needle, and bars represent mean ± SD. Mann–Whitney *U* test was used to test for differences between groups. (**H**) Dorsal (top panel) and lateral view (bottom panel) of embryo development progress from single-cell stage (0.5 hpf [hours post-fertilization], two-cell stage (2 hpf), to four-cell stage (3 hpf). (**J**) Embryo development from 2 dpf (days post-fertilization) to 5 dpf. Note that eumelanin pigmentation on yolk appears at 2 dpf; pigmented optic cups with lens placodes become discernible at 4 dpf; tail region separates from yolk (hatching) by 5 dpf.

Generation of knockout or transgenic lines requires a reliable source of embryos. We designate several 80–120 L tanks that house a single sexually mature male and a cohort of sexually mature wildtype females, separated by a barrier to control the onset of spawning (Fig. 3A). Because *A. burtoni* have an approximately 1-month long ovarian cycle^27^, we include sexually mature females in sufficient numbers (10–20) to increase the likelihood of a spawning. We have historically observed a high, but variable rate of embryo death after injection of CRISPR components. We hypothesized that standardizing the method by which embryos are manipulated prior to injection and/or irregularities in microinjection needles may contribute to this variability. First, we designed a 3D-printed mold for an agarose embryo holder to secure the embryos during injections (Fig. 3B,C). This reduced and standardized manual handling compared to prior approaches^28^. This had the benefit of improving speed of embryo injection by ~ 2-fold. We find that embryo survival in the agarose wells is comparable to unhandled embryos (survival rate for uninjected embryos in beaker, 67 ± 4%; in wells 63 ± 4%; *p* = 0.363, Student’s t test).

Microinjection needles are generated from glass capillary tubes (Fig. 3D,E) and breakage yields needles of variable size. Variation in microinjection needles could impact both the volume of reagents injected as well as the extent of damage to the embryo. To test whether this variation contributes to embryonic death rate, we screened needle widths, and correlated these to survival rate. We found that bores larger than 12.5 µm are associated with lower post-injection survival rates (Fig. 3G). Thus, needle widths from 7.5 to 12.5 µm permit maximal survival. Other factors associated with needle shape could also contribute to embryo death rate. For example, the angle or unevenness of the needle tip breakage (Fig. 3E) could affect death rate, but these were not quantified. Punctured embryos may release some contents of the yolk after injection (Fig. 3F). We observed that leakage of yolk during injection often results in low survival (Fig. 3I), which may be due to internal pressure caused by large embryos fit into small holes in the injection mold. Hence, we designed our injection mold with varied sizes of pegs of increasing diameter from 1.81 to 2.13 mm, allowing us to fit each embryo into an appropriately sized well.

### Manipulation of the cichlid Tyrosinase gene

**Figure 4:**
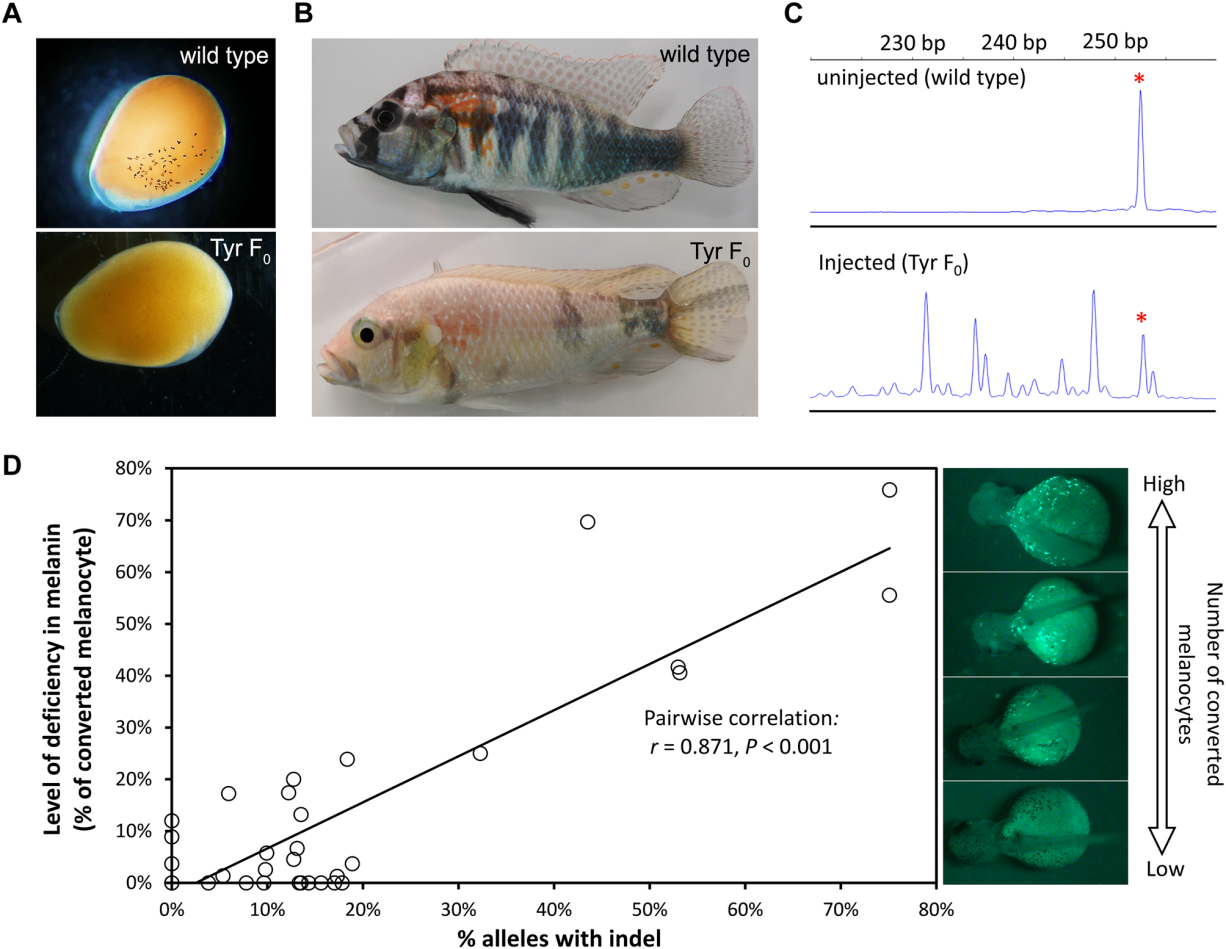
Analysis of parameters that affect CRISPR mutation rate is facilitated by *Tyr1* targeting. The contrasting melanin phenotypes of male wildtype (wt) and *Tyr1* gRNA-injected (*Tyr* F_0_) animals is visible from (**A**) 2–3 dpf to (**B**) adulthood. We note a 0.5–1.0 day developmental delay for the onset of pigmented melanocyte in CRISPR/Cas-injected embryos, regardless of gRNA target. (**C**) PCR fragment analysis reveals the extent of indel mutations: uninjected animals exhibit a single peak (denoted by asterisk). CRISPR/Cas injection induces indel mutations, resulting in peaks at varying sizes indicative of independent mutation events occurring in multicellular embryos and a resultant mosaic mutation pattern. Peak sizes are proportional to allele frequency, enabling calculation of mutation efficiency. (**D**) F_0_ animals are mosaics of cells carrying mutated and intact Tyr1 alleles, resulting in splotched pigment pattern. The rate of indel at *Tyr1* correlates with the conversion of melanocytes from black to fluorescent.

**Figure 5:**
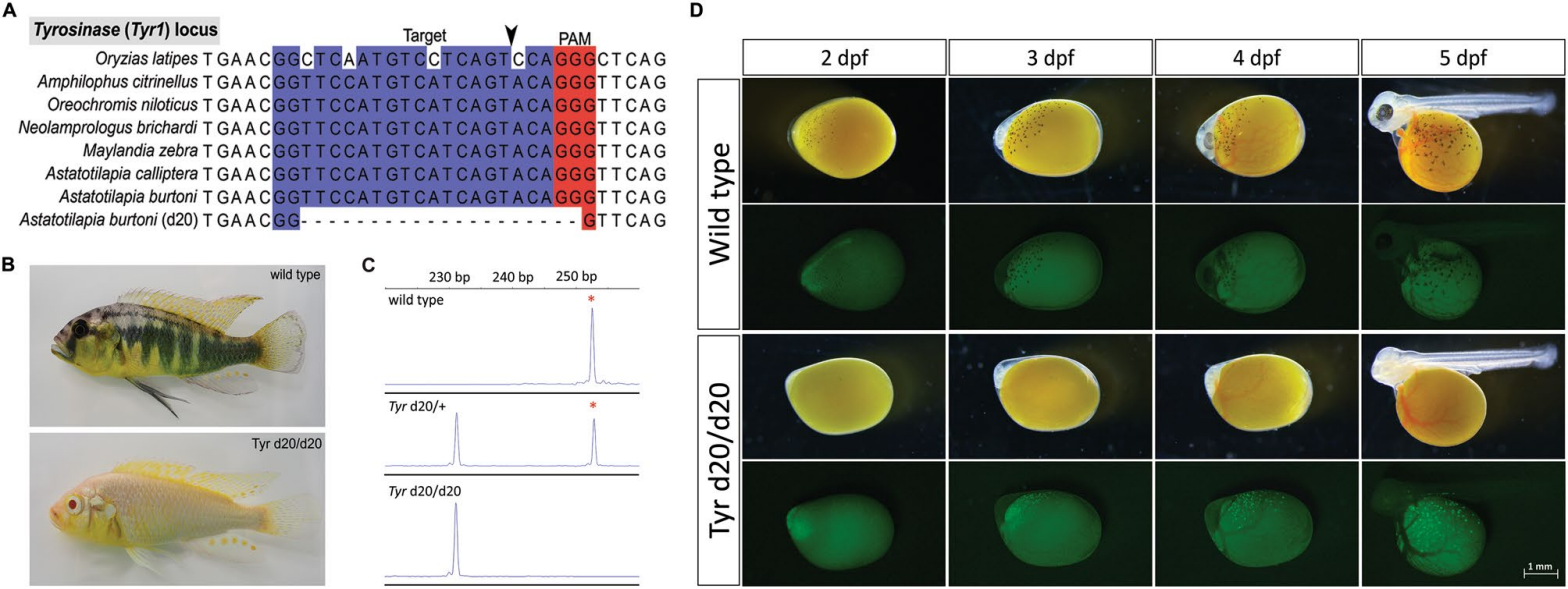
Creation of *Tyrosinase* mutant *A. burtoni*. (**A**) The CRISPR target site within the *Tyr1* locus is conserved across cichlid species. gRNA binding sequence is in blue; protospacer-adjacent motif (PAM) is in red. Arrowhead indicates the site of Cas9-mediated double strand break. *Astatotilapia burtoni* (d20) denotes a CRISPR/Cas-induced 20 bp deletion in *A. burtoni*. (**B**) Adult *Tyr1*^d20/d20^ animals (bottom) exhibit no eumelanin compared to wild type animals (top). (**C**) PCR fragment analysis shows that *Tyr* heterozygous mutants (*Tyr1*^d20/+^ among F_1_ offspring) exhibit one additional peak resulting from indel mutation (middle) compared to wild type (top); while *Tyr* homozygous mutants only have one peak corresponding to a 20 bp deletion (bottom). Wild type peak is denoted by asterisk. (**D**) *A. burtoni* homozygous for *Tyr1*^d20/d20^ mutation exhibit a loss of pigment in eye as well as neural crest-derived melanocytes that cover the yolk and body. This phenotype is observable starting from 2 dpf (Top panel). The converted melanocytes which exhibit green fluorescence are also observed starting from 2 to 3 dpf (bottom panel).

The ease of observing pigment cells early in development implies that a manipulation of their development or function would provide a platform for screening gene editing parameters. The *Tyrosinase* (*Tyr1*) gene has been shown to be necessary in medaka, zebrafish, and anole lizards for producing eumelanin from tyrosine^24,25,29^. We synthesized gRNAs that target the intramelanosomal, common central domain of *Tyr1*, a sequence identical at the nucleotide level across cichlids (Fig. 5A). We injected these along with Cas9 protein into single-cell embryos (Fig. 3H). We monitor embryo survival for approximately 10 days post-fertilization (dpf) to identify issues that contribute to low survival rates (Fig. 3J). By 3 days post-fertilization, uninjected embryos developed black, ramified melanocytes. In contrast, we found that ~ 50% of injected embryos exhibited a reduction in the number of melanocytes (Fig. 4A), suggesting that *Tyr1* gene function is necessary for eumelanin synthesis in cichlids.

We were surprised to find ramified, fluorescent cells scattered across the surface of *Tyr1* CRISPR-injected cichlid embryos, but not in control embryos (Fig. 4D). These cells also appeared at 2–3 dpf, and their number was inversely proportional to the number of black melanocytes. This suggests that in the absence of functional Tyr1, melanocytes accumulated a fluorescent metabolite derived from tyrosine. Further, we speculate this is not tyrosine itself due to its green, rather than blue, fluorescence emission^30^. As CRISPR-injected embryos are mosaics of cells bearing different mutations or unmodified alleles, the conversion of melanocytes from black (Tyr-positive) to fluorescent (Tyr-mutant) permits a quantification of gene editing efficiency. To test whether this metric reflected genomic mutation rate, we quantified the ratio of melanocyte conversion and obtained genomic DNA from embryos to assess indel rates. We adapted a PCR amplification approach^31^ to evaluate mutation induction in injected embryos using PCR size analysis. This approach quantitatively and rapidly detects size polymorphisms that result from indel mutations (Fig. 4C), the sequence changes most likely to affect gene function. We found a positive correlation between indel rate and black-to-fluorescent melanocyte conversion (Fig. 4D). Notably, melanocytes can be rapidly counted, and can be performed at an early stage. It thus will enable a rapid exploration of CRISPR parameters, and to troubleshoot problematic reagents.

Previous work using CRISPR/Cas to generate mutant cichlids utilized single guide RNAs (sgRNAs) that incorporate both crRNA and tracrRNA^32–34^. Recent work shows that using separate crRNA and tracrRNA as is utilized naturally in *S. pyogenes* (dual-guide RNA system; dgRNA) drives more efficient gene editing in zebrafish than does sgRNA^35^. We synthesized sgRNA and dgRNA (Supplementary Fig. 1A) that target the same *Tyr1* site to directly test whether this finding holds true in cichlids. We find high gene-editing rates at the *Tyr1* locus with either sgRNA or dgRNA systems (Supplementary Fig. 1B).

### Homozygous Tyr1 mutant cichlids enable in situ imaging

*Tyr1* CRISPR-injected fish remain mosaic animals through adulthood (Fig. 4B). We crossed injected fish to wild-types and screened offspring using *Tyr1* locus-specific fragment analysis. We identified a fish heterozygous for a 20 bp deletion at *Tyr1* (*Tyr1*^d20/+^) (Fig. 5C). An intercross of *Tyr1*^d20/+^ animals yielded 1/4 of offspring devoid of eumelanin from 2 dpf through adulthood (Fig. 5B). In place of black melanocytes, fluorescent cells can be readily observed from 2 to 10 dpf (Fig. 5D). The eumelanin deficiency remains a stable into adulthood (Fig. 5B), implying that developmental patterning signals from melanocytes remain intact^36^.

**Figure 6:**
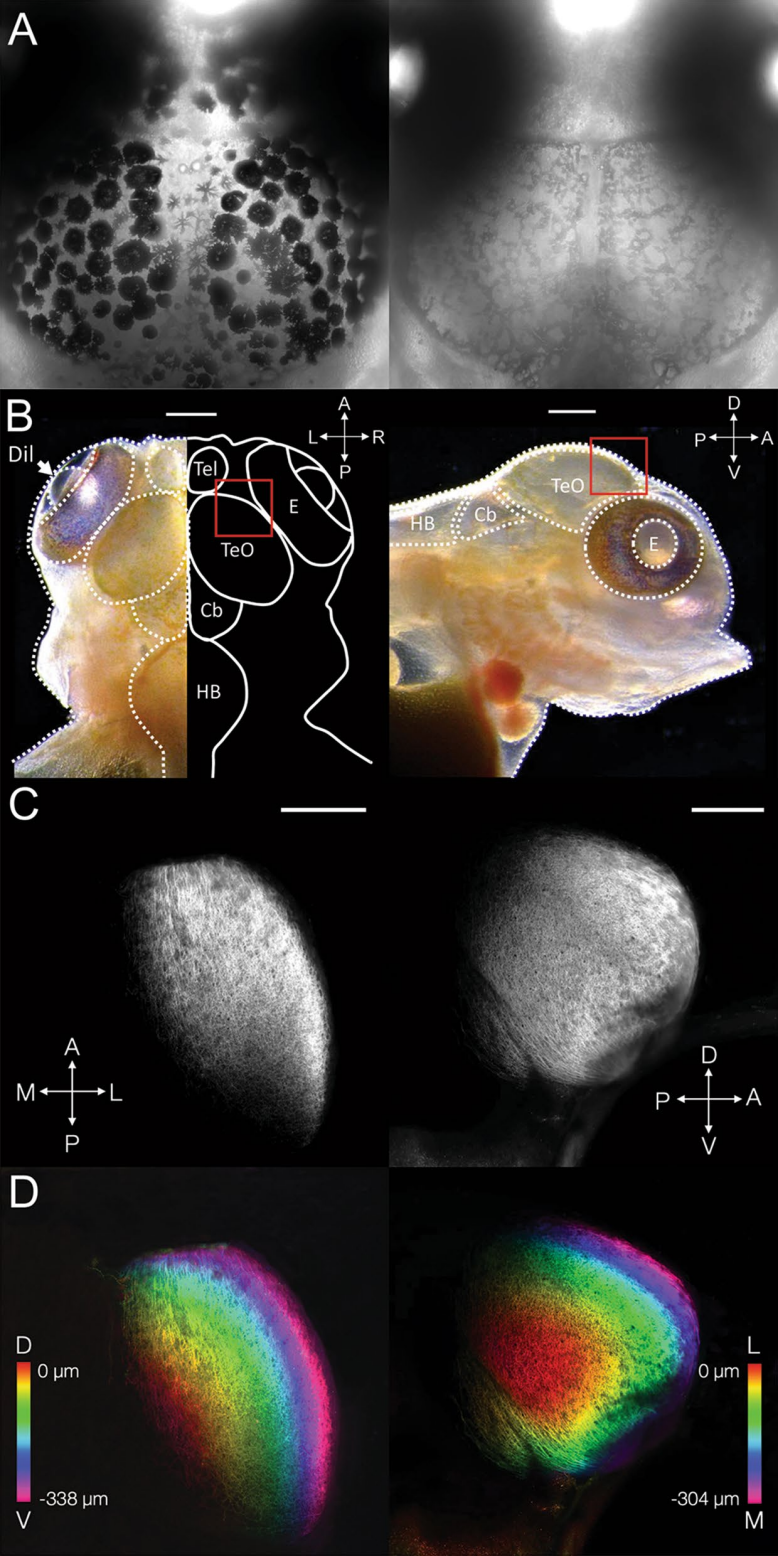
*Tyrosinase* mutant *A. burtoni* permit brain imaging in situ. (**A**) Dorsal view of the skin covering the midbrain in wild-type (left) and *Tyr1*^d20/d20^ (right) fish at 9 dpf, imaged using transmitted light. Pigmented melanocytes are absent above the brain in *Tyr1*^d20/d20^ fish, enabling underlying imaging of brain structures. (**B**) Dorsal view (left) and lateral view (right) of the head structure with schematic brain illustrations in *Tyr1*^d20/d20^ larvae at 9 dpf. Note that in the DiI injection experiment, those images were focused on dorsal and lateral edges of optic tectum (regions in red squares). *E* eyes, *Tel* telencephalon, *TeO* coptic tectum, *Cb* cerebellum, *HB* hindbrain. (**C**,**D**) Contralateral retinotectal projections were visualized by confocal microscopy in 9 dpf *Tyr1*^d20/d20^ cichlids after unilateral eye injection with DiI. Z-projections from dorsal (338 µm depth) (**C**,**D**, left) and lateral (304 µm depth) aspects (**C**,**D**, right) reveal retinal ganglion cell axons terminating throughout the peripheral optic tectum. Different colors depict the depth of brain tissues from the center surface (red) to the edge of the optic tectum (magenta). Scale bars 200 µm.

The absence of eumelanin in *Tyr1*^d20/d20^ cichlids also permits unobstructed visualization of subdermal features (Fig. 6A,B). As proof-of-principle, we unilaterally injected one eye of larval cichlids with the lipophilic fluorescent tracer DiI to label retinal ganglion cell (RGC) projections to the brain. After 3 days of diffusion, we imaged the optic tectum, to visualize RGC targets in situ. Imaging revealed dense RGC axonal arbors that wrapped around the dorsal and lateral edges of the tectum contralateral to the injected eye (Fig. 6C,D).

## Discussion

Many fundamental insights about evolution, development, physiology, and medicine have been derived from the study of organisms that are not regularly genetically modified. The advent of the CRISPR/Cas system enables reverse genetic approaches in species beyond traditional species such as *Drosophila* and mouse. Our protocol utilizing this system efficiently edits the genome in the non-traditional model species, *A. burtoni*. This African cichlid is a model organism for studies from behavioral neuroscience to ecology and evolution; CRISPR/Cas provides genetic insights into mechanisms of development and physiology. We targeted *Tyr1* gene as an example, and further demonstrated that CRISPR/Cas9 editing rates at the *Tyr1* locus as determined by tissue biopsy are correlated with the deficiency in melanin. The mechanism by which the mutation leads to the loss of pigmentation remains unclear; while we suggest a frameshift 5’ to a critical copper binding domain is causative, alterations in splicing could contribute. Nonetheless, the externally visible phenotype can be screened in the embryos as early as 2–3 dpf, which makes the *Tyr1* gRNA-injection a fast and reliable tool to quickly validate CRISPR/Cas activity in vivo, and to troubleshoot problematic reagents and protocols.

*Tyrosinase* mutant animals will be broadly useful as models for in vivo neural imaging. Zebrafish (*Danio rerio*) are commonly used as models for live neural imaging in a variety of contexts due to the transparency of larvae^37–40^. Further, recent advancements in gene editing have facilitated the development of novel genetic lines that circumvent the interference of skin pigmentation in adults^39,41,42^. Our *Tyr1*^d20/d20^ line also lacks melanocyte pigmentation, opening the prospect of in vivo neuroimaging in *A. burtoni*. Our DiI tracer injections demonstrate the ability to map the neural arborizations of retinotectal connections in an intact cichlid. These approaches have the potential to reveal important insights on the neural pathways of information processing and evolutionary differences across species. Beyond simply facilitating development of CRISPR/Cas9 protocols, the *Tyr1* mutation provides an opportunity for exploration of behavior and neurophysiology. *A. burtoni* males participate in a complex social hierarchy in which dominant and subordinate males differ in pigmentation, physiology, and reproductive opportunities^14^. One of the starkest distinguishing features of dominant males is the “eyebar”: a vertical line of melanin-rich cells across the cheek (Fig. 5B, top). Melanosomes in eyebar cells rapidly disperse and contract, enabling dynamic responses to social stimuli, and is thought to be used as a signal between rivals^43,44^. Because *Tyr1*^d20/d20^ individuals lack all eumelanin (Fig. 5B, bottom), use of mutant males in territory defense assays enables manipulation of this signaling between males. Similarly, *Tyr1*^d20/d20^ males will make it possible to determine whether females rely on these pigmentation patterns as a mate quality cue.

This protocol for using CRISPR is well-suited to creating loss-of-function mutations, but there is also a need for efficient, targeted introduction of specific gene editing by knock-in mutations^45^. This approach can test how a genomic region has evolved to differentially regulate a phenotype across species by transferring a sequence from one species to the orthologous locus in another. Knock-in mutations also enable the expression of a transgene using the complete gene regulatory environment of a locus, permitting faithful recapitulation of transgene expression. Such a system would enable conditional gene manipulation as well. For example, the Cre/*loxP* system enables site-specific or temporally delimited mutations^46^, but *loxP* sites must be inserted into the genetic locus of interest, and Cre lines benefit from the faithful gene expression described above. Progress has been made in targeted sequence insertions^19,20,22^, aided by chemical treatments and specific sequence features that increase HDR efficiency^47–50^. Homology-independent approaches for sequence knock-in may be more attractive given natural biases towards NHEJ over HDR^51^, and have demonstrated success in zebrafish and medaka^20,52,53^. Testing for repair of the mutated *A. burtoni Tyr1* locus will provide a platform on which cichlid knock-in experiment conditions may be rapidly optimized. Correct insertion of a repair template will restore pigmentation in the *Tyr1*^d20/d20^ fish, providing a visible, quantifiable readout of gene modification. As an alternative to genome editing by knock-in to obtain conditional mutants, transposon-based systems have the potential to permit cell-type specific manipulations. Prior work in cichlids has shown that the *Tol2* transposon system efficiently catalyzes transgene insertion and permits cell-type-specific transgene expression^28,54–58^. This system could be adapted to express CRISPR components using cell type-specific promoter sequences, thereby facilitating spatially restricted mutagenesis^59^.

A major advantage of cichlids as research models is the diversity of form and function across species. However, benefits accrue to research communities from a focus on a limited number of species. This allows the sharing of transgenic or CRISPR-edited lines, leveraging the size of the community to increase the number of experiments possible using existing lines. Furthermore, a focus on a limited set of species increases the available experimental tools, bioinformatic resources, and more^17^. *A. burtoni* is a well-suited model system as there is already a wealth of data and experimental protocols developed. In addition, as a basal *Haplochromine* cichlid, it is an ideal species with which to test hypotheses generated in the speciose radiations of *Haplochromines* in Lakes Malawi and Victoria. Thus, genetic engineering in *A. burtoni* promises to reveal novel genetic mechanisms for phenotypic diversity in behaviors, physiology, and anatomy.

## Methods

### Animals

Fish were bred and used at the University of Maryland from a colony derived from Lake Tanganyika^60^, according to the guidelines of the University of Maryland animal care and use committee. Tanks received recirculated water with constant pH (8.0–8.2) and salinity (320–480 ppm). Fish were housed in 30 L tanks prior to embryo collection, then in 6-well plates and 1.2 L tanks during growth. All animal maintenance and experimental procedures were approved by the Institutional Animal Care and Use Committee of the University of Maryland (protocol 1046257-1). The study was carried out in compliance with the ARRIVE guidelines.

### Fecundity testing

For each trial, we selected two size-match adult females (mass differences < 1.0 g), and each was injected intraperitoneally with either Ovaprim (Syndel) or saline at 0.5 μL per gram of body mass at 10 days after previous spawning. We then tagged injected females with visible elastomer tags (Northwest Marine Technologies) to distinguish treatments. After injection, fish were monitored in an isolated 2 L tank 30 min before returned to community housing. These females were co-housed with a dominant male which was separated by a transparent divider. We removed the divider for 30 min and monitored females for spawning behavior daily across the following two reproductive cycles, until they were observed carrying a brood. Embryos were collected 30 min after initial spawning and fertilization, and their survival was tracked until 12 dpf.

### CRISPR injections

Microinjection needles are generated from glass capillary tubes (GC100F-10, 1.0 mm O.D; 0.58 mm I.D; Harvard Apparatus) by first using a micropipette puller (Sutter, P-97) to separate the tube into two needles and then opening the tip by gently tapping the needle on a taut Kimwipe to break the tip to ~ 10 µm diameter (see Supplementary Methods *Microinjection of Cichlid Embryos* for detail). Needle bore width was defined as the long axis of the needle’s angled opening (not the distance orthogonal to the axis of the tube). Resulting needles are screened by microscopy to confirm sizes are ≤ 12.5 µm. All oligonucleotides were ordered from IDT; crRNA and tracrRNA were synthesized with AltR modifications. sgRNA was synthesized as in^31^ by annealing an oligo with gene-specific sequence Tyr1 sgRNA2 with a universal lower oligo (Table 1). Taq polymerase (NEB) is used to extend dsDNA, and T7 RNA polymerase (NEB HiScribe kit) transcribes sgRNA (see Supplementary Methods *Design and Synthesis of gRNA* and *Cloning-free single guide RNA synthesis* for details). *Tyr1* crRNA and tracrRNA sequence (IDT; 1072532) are diluted to 25 µM in IDT duplex buffer. crRNA:tracrRNA duplex was prepared as in^35^. We combined components with the following final concentrations: Cas9 protein (Invitrogen, A36497; or IDT, Alt-R S.p. Cas9 Nuclease V3, 1081058) at 5 µM with Texas Red Dextran (Life Technologies, MW 3000 Da) at 0.5% and guide RNA(s) at 5 µM. At 30 min following the initiation of egg laying, embryos were collected using a transfer pipette to flush the mouthbrooder’s oral cavity with antifungal-treated tank water (see Supplementary Methods *Obtaining Cichlid Embryos* for details). For injection, eggs were placed into wells created by a mold with circular indentations (diameter from 1.81 to 2.13 mm) in 2% agarose made with tank water. Microinjections were performed using a stereomicroscope (Nikon, SMZ745) with a 3D micromanipulator (Narishige, M-152) and a source of pressurized air (Airgas, compressed dry air, size 200, adapter CGA-590) with a Milli-Pulse Pressure Injector (Applied Scientific Instrumentation, MPPI-3). The cell of each embryo was targeted and injected with two pulses of approximately 1 nL of solution (each 2.5 ms at 22 psi). Embryos were monitored for leakage of yolk during injection and daily survival to 12 dpf. See Supplementary Methods *Microinjection of Cichlid Embryos* for more detailed procedures.

**Table 1:** Oligonucleotides used in the experiments.

Oligonucleotide name	Sequence (5'–3')	Notes
TyrFlankF-M13	TGT AAA ACG ACG GCC AGT cag gtt ttg caa gtc cac aga c	M13 binding site in uppercase
TyrFlankR-pigtail	GTG TCT Ttc ttt ctc act gca tta cac cc	Pigtail sequence in uppercase
Tyr1 sgRNA2	TTA ATA CGA CTC ACT ATA ggt tcc atg tca tca gta caG TTT TAG AGC TAG AAA TAG C	Tyr1 binding sequence in lowercase
Universal lower oligo	AAA AGC ACC GAC TCG GTG CCA CTT TTT CAA GTT GAT AAC GGA CTA GCC TTA TTT TAA CTT GCT ATT TCT AGC TCT AAA AC	
Tyr1 crRNA	/AltR1/rGrG rUrUrC rCrArU rGrUrC rArUrC rArGrU rArCrA rGrUrU rUrUrA rGrArG rCrUrA rUrGrC rU/AltR2/	
M13-Fluorescein	/56-FAM/TG TAA AAC GAC GGC CAG T	

### Indel mutation quantification

We obtained genomic DNA by removing ~ 1 mm^3^ of fin tissue, added 180 µL of 50 mM NaOH, and incubated at 95 °C for 15 min. Samples were neutralized with 20 µL of 1 M Tris (pH 8.0). We PCR amplified the region spanning the CRISPR target site of *Tyr1* using TyrFlankF-M13, TyrFlankR-pigtail, and M13-Fluorescein (Table 1). Fluorescein-labeled amplicons were separated and quantified during capillary electrophoresis (3730xl DNA Analyzer, Applied Biosystems; see Supplementary Methods *Quantifying mutation prevalence and identifying mutant cichlids* for details). Peak prevalence relative to wild type was analyzed using Peak Scanner and fragmentanalysis.com. Briefly, we quantified allele frequency within the mosaic tissue by using Peak Scanner to measure the area under peaks of each amplicon length. The ratio of the area under the wildtype peak to sum of areas under all peaks represents the fraction of unmodified alleles.

### Melanocyte quantification

To quantify black and fluorescent melanocytes, *Tyr1* gRNA injected embryos at 5 dpf were chilled in ice-cold tank water for 30 s to immobilize them, and then were transferred to a 6 well plate to detect green fluorescence under a stereomicroscope (Nikon, SMZ18). We used a soft paintbrush (Robert Simmon, size 1) to flip the embryos to ensure that we counted all melanocytes. After melanocyte quantification, we obtained genomic DNA by removing ~ 1 mm^3^ of posterior tissue from each embryo for fragment analysis for assessing indel rates. The level of deficiency in melanin was calculated as the number of fluorescent melanocytes divided by the number of total melanocytes (Fig. 4). We further categorized these *Tyr1*-injected embryos by pigmentation phenotypes using a fluorescence stereomicroscope (Leica M165 FC) using band-pass filter cubes for the detection of green fluorescence (ET GFP-M205FA/M165FC Excitation: 470/40, Emission: ET525/50). If the percentage of fluorescent (converted) melanocytes cells is smaller than 10%, the embryo is categorized as ‘normal pigmentation’; if the percentage of fluorescent melanocytes cells is between 10 and 50%, then it is categorized as ‘sparse pigmentation’; if the percentage of fluorescent melanocytes cells is larger than 50%, then it is categorized as ‘significantly diminished pigmentation’ (Supplementary Fig. 1B).

### Retinotectal projection labeling

We fixed larval cichlids at 9 dpf overnight in 4% paraformaldehyde and washed them in PBS. DiI (Life Technologies V22889) was pressure injected to fill the eye cavity unilaterally and allowed to diffuse for 3 days at 28 °C. We imaged tectal RGC arborizations in situ using a Zeiss LSM800 confocal microscope and a 10 × 0.5NA objective.

### Images

The graphics of *A. burtoni* used in Figs. 1, 3, and Supplementary Fig. 1A were drawn in Adobe Photoshop (version 22.4.2).

## Supplementary Information

Supplementary Information 1.
