# Supplementary material for: Manipulation of the Tyrosinase gene permits improved CRISPR/Cas editing and neural imaging in cichlid fish
Source: Sci Rep. 2021 Jul 23;11:15138. doi: 10.1038/s41598-021-94577-8 (PMC8302579; doi:10.1038/s41598-021-94577-8)
Supplement: Supplementary file 1 — Supplementary Information 1. [file 41598_2021_94577_MOESM1_ESM.pdf]

Supplementary Methods

Design and Synthesis of gRNA

The most important step in recovering LoF mutations is to select a CRISPR target site that will abolish the function of a key protein domain(s), so knowledge of the functional domains of the gene is desirable. It is key to understand the genetic structure of the gene including alternative start sites, alternative exons, and the location of functional domains. Analysis using NCBI or Ensembl genome browsers for cichlids and other model species should allow for identification of these features. We typically target a site 5' to the necessary functional domain(s) and seek animals carrying a frameshift mutation that causes a failure to correctly encode amino acid residues 3' of the mutation. In the absence of *a priori* knowledge of functional domains, it is advisable to introduce a frameshift mutation near the 5' end of the gene to maximize the likelihood of generating a missense translation of a large portion. There are several nuances to this approach, however. First, alternative translational start sites and alternative transcript isoforms are common. A mutation in the 5' end of the gene may not result in protein sequence changes if translation is initiated at a site 3' of the mutation. Similarly, if alternative transcripts exist for the gene (i.e. splice variants or alternative transcription start sites), a functional protein may still be produced from the gene. Second, mutations near the 5' end of the transcript may result in short open-reading frames, thereby triggering nonsense-mediated decay and the upregulation of a paralogous gene(s)[1,2]. In general, after creation of mutant lines, it is important to determine whether mRNA levels are altered for paralogous genes.

CRISPR/Cas targeting is not equally effective at all sites. The CRISPR protospacer-adjacent motif (PAM) NGG site of the *Streptococcus pyogenes*-derived Cas9 enzyme institutes a requirement for a GG dinucleotide sequence just 3' to the double-stranded break site. This requirement can be altered or relaxed by using alternative Cas proteins (e.g., Cpf1)[3] or engineered Cas9 [4], but a GG sequence can be found in the coding sequence of most genes. There are additional sequence parameters that modulate the efficiency of cutting, and a variety of web tools are available for identifying CRISPR target sequence[5]. CHOPCHOP (https://chopchop.cbu.uib.no) [6] identifies sites in numerous species (including cichlids), and predicts efficiency of on-target mutations and rate of off-target mutations. The program provides an interactive graphical representation of the gene with each potential target and splice isoform. We recommend selecting two target sites, separated by ~50 bp. This increases the chance of introducing a mutation, and in some cells

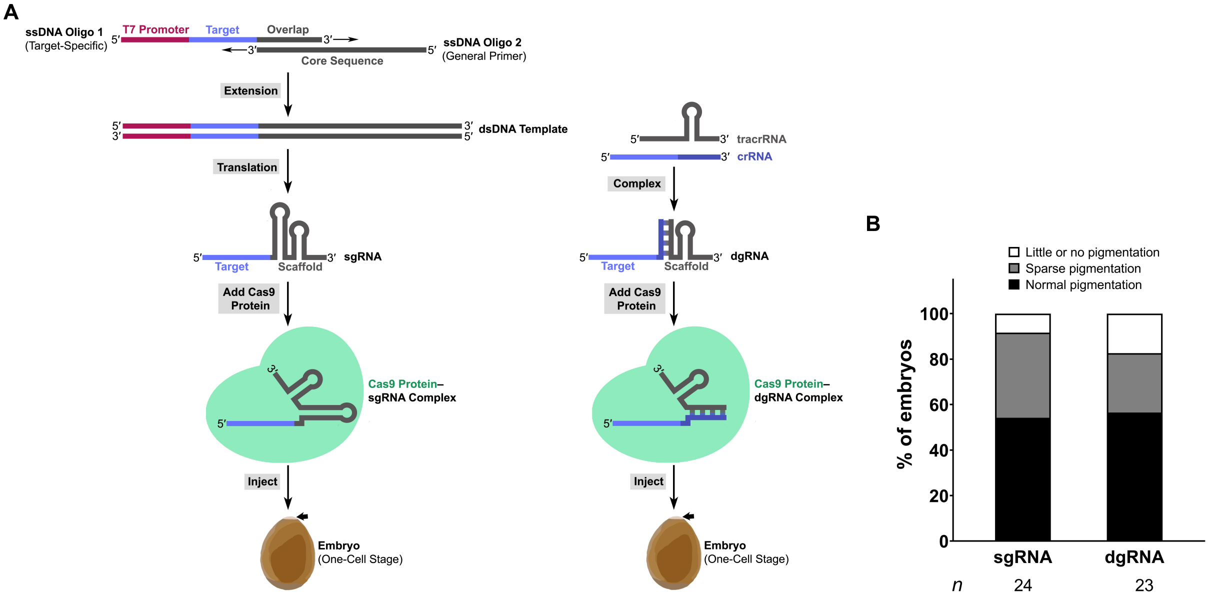

Supplementary Figure 1. Generation and comparison of single guide RNA versus dual guide RNA. (A) Single guide RNA (sgRNA, left) is generated by annealing two partially complementary single-stranded oligonucleotides (ssDNA), then extending into double-stranded DNA (dsDNA). T7 polymerase binds the T7 promoter sequence and transcribes the crRNA (gene-specific) and tracrRNA (universal) components, connected by a hairpin sequence. The dual guide RNA (dgRNA) system (right) provides the tracrRNA and crRNA sequences in *trans*, and these associate by base pairing. For each, the guide RNA(s) are incubated with Cas9 protein to form ribonucleoprotein complexes for embryo injection. **(B)** Induction of mutations in the *Tyr1* locus using sgRNA or dgRNA is similarly efficient. The percentage of embryos with pigmentation that is normal, sparse, or lost altogether at 5 dpf using sgRNA or dgRNA in ribonucleoprotein complexes. N = 23-24 embryos across 4 independent injections for each type of gRNA.

both sites will be cut simultaneously, resulting in a deletion of ~50 bp[7]. A larger deletion increases the likelihood of loss of function and permits easy detection when genotyping future generations by PCR.

In the process of creating mutations, the variety of indels generated for most cut sites is unpredictable. However, we have encountered cut sites that commonly produce the same mutations, likely as a result of robust microhomology-mediated end joining (MMEJ) repair. While this may result in more reproducible phenotypes in injected fish[8], it may also result in the production of non-frameshift mutations at high rates. We use MENTHU (Microhomology-mediated End joining kNockout Target Heuristic Utility; http://www.genesculpt.org/) [9] to avoid microhomology-mediated end joining allele repair outcomes with predicted weak effects (e.g., indels of 3, 6, or 9 base pairs).

Guide RNA systems take one of two forms: 1) The naturalistic dual-guide RNAs (dgRNA) in which a trans-activating CRISPR RNA (tracrRNA) and CRISPR RNA (crRNA) are provided as separate components, or 2) A single guide RNA (sgRNA) that consists of a single combined crRNA and tracrRNA construct. We utilize a cloning-free, cost-effective method to synthesize sgRNA which is adapted from (Varshney et al., 2016, **Supplementary Figure 1A**). Alternatively, synthesized CRISPR dgRNA components may be ordered commercially: a target-specific crRNA and universal tracrRNA (Alt-R CRISPR-Cas9 tracrRNA, IDT). Though most of our mutant lines have been generated using sgRNA and we find high mutation rates in *A. burtoni* using sgRNA, researchers should consider the dgRNA approach because work in zebrafish has been shown it improves editing rates[11]. We find that the dgRNA approach works well in our hands. Additionally, dgRNA can be synthesized commercially for <$100 each and the time savings are significant.

Cloning-free single guide RNA synthesis

We use a cloning-free approach to synthesize sgRNAs, adapted from Varshney, Burgess and colleagues[10,12]. We use a target-specific oligonucleotide and a universal oligonucleotide that encodes the tracrRNA. A target-specific DNA oligo consists of the T7 RNA polymerase promoter, 18-20 bp target sequence, and 20 bp encoding a portion of the tracrRNA: (5'-TAA TAC GAC TCA CTA TA - $N_{18\text{-}20}$ – GTT TTA GAG CTA GAA ATA GC-3'). Note that the NGG PAM sequence is not included in the gRNA. The 20 bp at the 3' end are complementary to the universal oligonucleotide, permitting hybridization, extension, and amplification. A GG dinucleotide is required at the 5' end of the gene-specific sequence for the initiation of transcription by T7 polymerase. Thus, utilizing a target sequence that contains GG at its 5' end satisfies this requirement. Alternatively, 1-2 guanines not complementary to the target sequence may be added to the 5' end of the gRNA, though this may hinder mutation rate[11]. As an example, the target-specific oligo we use to target the eumelanin-synthesis enzyme *Tyr1* gene is 5'-TTA ATA CGA CTC ACT ATA ggt tcc atg tca tca gta ca GTT TTA GAG CTA GAA ATA GC-3', where sequence specific to *Tyr1* is in lowercase. The universal DNA oligo is an 80 bp sgRNA core sequence: (5'-AAA AGC ACC GAC TCG GTG CCA CTT TTT CAA GTT GAT AAC GGA CTA GCC TTA TTT TAA CTT GCT ATT TCT AGC TCT AAA AC-3'). Oligonucleotides may be ordered from commercial providers (eg, IDT). The universal oligo should be purified (eg, by PAGE) to ensure a full-length oligonucleotide. Here is our protocol for synthesizing single guide RNA:

1. Dilute oligos to 10 µM
2. Combine the following:
   - 19.5 µl ultrapure DI H_2O
   - 2.5 µl 10x Standard Taq Polymerase Buffer (NEB)
   - 0.5 µl dNTP (10 mM)
   - 1 µl Oligo 1 (Target-specific; 10 µM)
   - 1 µl Oligo 2 (general; 10 µM)
   - 0.5 µl Taq (NEB)
3. On a thermocycler, anneal oligos as follows:
   94°C, 2 min; 50°C, 10 min; 72°C, 10 min; hold at 4ºC
4. Confirm presence of ~120 bp band on 2% agarose gel; band(s) <100 bp observed in controls without Taq polymerase.
5. Transcribe sgRNA:
   - 3 µl Annealed oligos
   - 5 µl ultrapure DI H_2O
   - 10 µl 2x NTP mix (NEB HiScribe T7 kit)
   - 2 µl T7 polymerase

Incubate at 37ºC for 2-16 hours.

6. Digest DNA template by adding 1 µl DNase (Ambion), incubate at 37ºC for 15 minutes
7. Precipitate RNA. Add:
   85 µl ultrapure DI H_2O O
   100 µl Isopropanol (100%)
   10 µl Sodium acetate (3 M, pH 5.2)
   Precipitate at -20ºC for ≥30 minutes.
8. Purify sgRNA:
   Spin at 4ºC for 15 minutes at ~16,000 x g, remove supernatant carefully and discard.
   Add 200 µl Ethanol (70%) to wash
   Spin for 5 minutes at ~16,000 x g, remove supernatant carefully and discard.
   Air dry for 5-10 minutes.
9. Resuspend pellet in 20 µl RNase-free water, pipet to mix.
10. Run 1 µl on 2% agarose gel; expect ~100 bp band.
11. Measure A260 concentration on a spectrophotometer.
12. Make ~1.5 µL aliquots at 1 µg/µL, store at -80ºC.

Obtaining Cichlid Embryos

Generation of knockout or transgenic lines requires a reliable source of embryos. We designate several 80-120 liter tanks that house a single sexually mature male and a cohort of sexually mature wildtype females, separated by a barrier to control the onset of spawning. Because *A. burtoni* have an approximately one-month long ovarian cycle[13], we include sexually mature females in sufficient numbers (10-20) to increase the likelihood of a spawning. Halved terra cotta flower pots serve as shelters and spawning sites for *A. burtoni*, but the ideal environment will be species-specific.

We find that Ovaprim injection shortens the time to egg laying by ~5 days. It also greatly increases the yield of eggs laid, without affecting egg viability. Interestingly, the increased fecundity effect of Ovaprim is observed in the following ovarian cycle, though period of the cycle returns to normal. Aside from such chemical treatments, it may be helpful to track individual females' reproductive activity. Moving a female to individual housing or labelling females with visible elastomer tags (Northwest Marine Technologies) after spawning makes it possible to monitor their progress through the reproductive cycle and predict future spawning.

On the desired day of injections, we inspect tanks to find females who are gravid with eggs (suggested by abdominal distension), aggressive or territorial, or have a protruding genital papilla. Females with these characteristics are generally the ones most likely to mate. We remove the barrier between females and the male and monitor the tanks for ≤30 minutes, the typical maximum time preceding spawning. During spawning, *A. burtoni* exhibit multiple rounds of egg laying and fertilization [14]. We therefore allow 30 minutes of spawning to maximize the number of fertilized eggs.

Before collecting eggs, we treat 1 liter of tank water with 1 mg/L methylene blue antifungal reagent (Sigma). We add 6 mL of the treated water to each well of six-well plates to house each embryo after injection. We find that use of well plates is very helpful as it permits alteration of experimental parameters and tracking the outcomes of individual embryos. Furthermore, the death of large cichlid embryos rapidly fouls water quality; the well plates separate healthy from unhealthy embryos. During spawning, CRISPR components for injection are also assembled (see below). After 30 minutes, fertilized eggs are collected by netting the mouthbrooding female and holding her above a beaker with treated tank water and using a narrow mouth transfer pipette (diameter ~2.5mm) to flush the mouthbrooder's oral cavity with water until all eggs have been collected. We use a transfer pipette cut to a wider diameter (~4 mm) to transfer eggs from the collection beaker.

Microinjection of cichlid embryos

Preparation. Microinjections require a standard stereomicroscope (Nikon, SMZ745) equipped with a 3D micromanipulator (Narishige, M-152), and a source of pressurized air (Airgas, compressed dry air, size 200, adapter CGA-590) equipped with a Milli-Pulse Pressure Injector (Applied Scientific Instrumentation; MPPI-3).

We designed an agarose embryo-holder to secure the embryos during injections, dramatically increasing throughput over prior work[15]. The mold is 3D-printed with acrylonitrile butadiene styrene (ABS) plastic filament, commonly used in 3D printers (print file available as Supplementary Material). The mold has 10 rows of 12 cylindrical pegs with rounded tops. Each row is comprised of 6 pairs of pegs of increasing diameter and height from 1.81 mm to 2.13 mm that are spaced 6 mm apart. This range in peg dimensions introduces variability in the size of wells in the gel, accommodating eggs either smaller or larger than the 2 mm typical of

A. burtoni embryos and maximizing the number of eggs that can fit securely within a single plate. The mold is placed inverted into 2% agarose solution (in tank water, boiled in the manner of a molecular biology agarose gel), poured into an appropriate container (we use the lid of a 6-well plate). The gel should be prepared at the beginning of the 30-minute window post-fertilization to give it time to solidify prior to injections. It may also be stored at 4ºC for ~1 week in a sealed plastic bag.

We create microinjection needles from glass capillary tubes (GC100F-10, 1.0 mm O.D; 0.58 mm I.D; Harvard Apparatus) pulled by a Sutter P-97 micropipette needle puller (Settings: heat, 515; pull, 60; velocity, 100; time, 170). This separates the capillary tube in two, with sharp, sealed tips. We break the tips by holding taut a Kimwipe and gently tapping the tip straight into it. We then screen the width of the needles' bores using a microscope equipped with an ocular micrometer. The ideal bore size of needles is between 7.5-12.5 µm, measured by the outer diameter. We find that bores larger than 12.5 µm are associated with lower post-injection survival rates. While we have not detected an impact of needle width on mutagenesis rates, we recommend trying a variety of sizes to confirm optimal survival and mutation rates.

In our experience, this diameter of needle delivers ~1 nL of solution when a 2.5 ms air pulse at 22 psi is delivered. We directly measure this volume by injecting 0.7% Phenol Red solution into a small petri dish of mineral oil (Sigma, M3516). The diameter of the resultant sphere of red solution can be measured under a microscope, and its volume calculated by $V = (4/3)*\pi r^2$. If the injection volume differs significantly from 1 nL, we recommend adjusting the air pulse duration.

<u>*Reagents*</u>. There are many acceptable suppliers and versions of Cas9 protein, but it is crucial that the protein contain a nuclear localization sequence to permit eukaryotic genome editing. We use Cas9 protein from Invitrogen (TrueCut Cas9 protein, A36497) or IDT (Alt-R S.p. Cas9 Nuclease V3, 1081058). We have also used an mRNA for zebrafish-codon-optimized *Cas9* expression carrying N- and C-terminal *NLS* sequences[14,16]. We find similarly high rates of editing with both protein and mRNA, but the ease and low cost of Cas9 protein makes it a good choice for creating loss-of-function mutations. Colored compounds like Texas Red-conjugated dextran or Phenol Red allow for easy visualization of the mixture during injection. Texas Red conjugated dextran (stock concentration 2.5%; Sigma, 3000) is helpful because its red fluorescence is retained through early development (~3 days), thus permitting visualization of the developing embryo and indication of gRNA/Cas9 delivery.

We have validated both sgRNA and dgRNA approaches in our genome editing protocol. The method to synthesize sgRNA is described in **Supplementary Figure 1A**. CRISPR dgRNAs were ordered from IDT. To prepare dgRNA for microinjection, we dissolve crRNA and tracrRNA as 100 µM stock solution in duplex buffer (30 mM Hepes pH 7.5, 100 mM Potassium acetate; IDT). To create a crRNA:tracrRNA duplex, mix equal volumes of crRNA and tracrRNA stock solutions and anneal on a thermocycler: 95°C, 5 min; cool at 0.1°C/sec to 25°C; 25°C, 5 min; cool to 4°C rapidly. This 50 µM crRNA:tracrRNA duplex stock is diluted with one volume of duplex buffer to produce a 25 µM working concentration. We also adjust Cas9 protein (Alt-R S.p. Cas9 Nuclease V3, 1081058) to 25 µM stock solution in 20 mM HEPES-NaOH (pH 7.5), 350 mM KCl, 20% glycerol. Our preliminary data in cichlids show high gene-editing rates at the *Tyr1* locus with either sgRNA or dgRNA systems (**Supplementary Figure 1B**). Work in zebrafish indicates that the dgRNA approach is more efficient than sgRNA[11]; future work at additional loci will determine whether this is true for cichlids as well.

<u>*Day of injection*</u>. After initial observation of egg laying, begin preparing reagents for injection while spawning continues. For CRISPR/Cas9 injection mixture, the reagents differ slightly depending on whether sgRNAs or dgRNAs will be used.

For sgRNAs, combine the following:

- 0.5 µL 100 ng/µL Cas9 protein (final conc. 100 ng/µL),
- 1.5 µL 1 µg/µL sgRNA (total) (final conc. 600 ng/µL),
- 0.5 µL 2.5% Texas Red conjugated dextran (final conc. 0.5%).

For dgRNAs, combine the following:

- 1.0 µL 25 µM Cas9 protein (final conc. 5 µM),
- 1.0 µL 25 µM crRNA:tracrRNA duplex (final conc. 5 µM),
- 2.5 µL ultrapure DI H_2O,
- 0.5 µL 2.5% Texas Red conjugated dextran (final conc. 0.25%).

Prior to microinjection, incubate the gRNA:Cas9 ribonucleoprotein complex solution at 37°C for 5 min and then backfill into ~3 microinjection needles using an Eppendorf GELoader tip (Eppendorf, 022351656). Label six-well plates to permit recording of protocol deviations and observations during injections.

Add tank water with methylene blue onto the agarose embryo holder to prevent desiccation of the embryos during injection. Collect embryos at 30 minutes after initiation of spawning. We recommend moving a moderate number (10-20) embryos directly into six-well plates instead of injecting them to serve as sibling controls. Some broods of embryos have low viability due to poor fertilization, genetic abnormality, or handling, and these controls help to distinguish from effects of injection or CRISPR components. Additional controls may be incorporated (see section "*Developing and troubleshooting cichlid gene editing*"). We use a wide-bore transfer pipette to transfer remaining eggs from the collection beaker to the agarose embryo holder and carefully guide them into the wells using a small paintbrush (Robert Simmon, size 1). Appropriate fit of embryos to the well is key. If the embryo is too small for the well, it may float out or be pulled out with the injection needle. If the embryo is too large, the pressure may force yolk out of eggs when pierced by the needle, resulting in low survival. We orient the eggs with cell mass directed upward to permit its injection; this is presumed to be more effective than injecting into the yolk[10]. However, we have observed successful editing following injection into yolk, presumably due to cytoplasmic streaming of maternal components from yolk to nucleus.

After loading embryos, we inject them with 2-3 pulses 1 nL, using 2.5 ms pulse duration at 22 psi. We gradually pull the needle out of the embryo during each pulse to reduce the local pressure. Note that the volume delivered to the embryo varies with the bore of the needle. As injections progress, the tip may break, resulting in large injection volumes and increased mortality. In this case, switch to a new needle loaded with injection mixture. We typically inject until embryos have reached the two-cell stage, visualized by a cell cleavage furrow approximately 90 minutes post-fertilization. Once injections are complete, embryos are gently removed from their wells using a micro laboratory spatula (Fisher, blade size: 19 mm × 4.8 mm) and placed individually in six-well plates with tank water containing methylene blue. While embryos develop, the plates are kept on an orbital shaker to facilitate oxygenation of water.

We monitor embryo survival for approximately 10 days post-fertilization to identify issues that contribute to low survival rates. For example, a failure to develop melanocytes (one of the earliest external markers of development), suggests that eggs may not have been properly fertilized. The ease of observing melanocytes early in development also makes *Tyr1* gRNA an attractive option for testing micro-injection efficiency. Furthermore, the sequence of the *Tyr1* gRNA target site is conserved across sequenced cichlid species, indicating that it will be useful in developing this technology for other species.

Quantifying mutation prevalence and identifying mutant cichlids

Because mutagenesis efficiency can vary widely, it is essential to develop a rapid method for evaluating mutagenesis efficiency. We adapted a PCR amplification approach [10] to evaluate mutation induction in injected embryos using PCR size analysis. This approach quantitatively and rapidly detects size polymorphisms that result from indel mutations, the sequence changes most likely to affect gene function. It does not identify missense mutations, but as these are less likely to lead to a loss-of-function mutation, we do not seek to quantify them. Another alternate genotyping approach is to select gRNA targets that disrupt a restriction enzyme recognition site[17]. PCR followed by restriction enzyme digest can identify embryos that become resistant to DNA cleavage after mutation of the target site. However, this approach relies on the presence of a restriction enzyme site at the target site, and is prone to false negatives since a mutation may be created that does not alter the restriction site. An alternative is the use of T7 Endonuclease I to cleave dsDNA that is imperfectly base paired, enabling discovery of embryos carrying alternate alleles[16,18]. While this is rapid, inexpensive, and generally useful, it is only semi-quantitative and does not reveal information about the nature of the mutation(s) introduced. The kinetics of DNA duplex melting may also be used to quantify the incidence of edited DNA using a quantitative PCR machine[19]. This approach rapidly identifies embryos carrying mutations, but does not predict the indel size.

Each approach relies on analysis of PCR amplicons that span the target site. CHOPCHOP suggests PCR primer pairs that flank each site which can be used to test for mutations. PCR amplification and sequencing using such primers should be performed prior to embryo injection to confirm that there are no naturally occurring polymorphisms at the locus that would affect gRNA targeting or genotyping before proceeding with injections. We sequence cut sites of ~5 unrelated animals, including breeding males, for each locus we target. This is important for most species as polymorphisms have not been purged through systematic inbreeding.

We PCR amplify the ~300 bp region surrounding the target using gene-specific primers. We append to the 5' end of the forward PCR primer an M13 sequence (5'- TGT AAA ACG ACG GCC AGT -3'), and a "pigtail" sequence to the 5' end of the reverse PCR primer (5'- GTGTCTT-3') to ensure terminal adenylation[20]. We use a common fluorescent M13 primer (5'-/56-FAM/TGT AAA ACG ACG GCC AGT-3', IDT) that tags PCR amplicons with fluorescein that can be detected after size separation using capillary electrophoresis (3730xl DNA Analyzer, Applied Biosystems). We then compare the PCR product size(s) of injected and un-injected individuals to determine the rate of genome modification.

We use a combination of approaches to determine the efficiency of key steps of gene editing. First, embryos that have been co-injected with Texas Red-conjugated dextran can be screened by fluorescent microscopy at 1 dpf to confirm that reagents were successfully injected, ruling out blockage of microinjection needles. We recommend beginning by targeting a gene that will yield an externally evident phenotype such as *Tyr1* mutation causing a loss of eumelanin. However, quantification of editing at most loci will require a genetic test. We use size analysis of fluorescent PCR products to identify indel mutations. Because extracting sufficient quantities of tissue for PCR in the first week of life is lethal, we sample only as necessary: we sacrifice 4-10 embryos at 5-7 dpf to confirm that the targeted locus is successfully mutagenized, and the remainder are allowed to grow to establish a genetic line. If the gRNA drives mutation at ≥50% of alleles, we consider it an efficient gRNA because these animals regularly transmit indel mutations to offspring. Unlike experiments in zebrafish where entire embryos are lysed, we sample genomic DNA by amputating the last third of an embryo's tail with fine forceps and placing each sample into 0.2 mL PCR tubes for lysis[21], a procedure facilitated by hatched embryos (4-5 dpf). In our experience, inclusion of the large yolk in genotyping samples leads to PCR failure. Future development of alternative protocols to inactivate or remove the problematic components will enable genotyping to occur at earlier stages. If the sampled embryos do not exhibit a high level of indel mutations at the target site, it implies that either the gRNA(s) selected are not effective or delivery of other reagents is compromised. To control for the activity of other reagents (eg, Cas9, tracrRNA) and injection problems, we rely on the injection of gRNA targeting *Tyr1* which exhibits the easily observable pigment loss. If gRNA against *Tyr1* works successfully, we then suggest proceeding to design new gRNAs against the target gene.

At ≥4 weeks of age, we finclip injected fish to identify those carrying the high rates of mutation. These fish with somatic mutations are preferred for breeding, as they are most likely to also carry mutations in the germline. We seek animals carrying >50% indel mutations for breeding, but have observed germline transmission from animals with indel rates of ~20%.

<u>DNA extraction</u> (modified HotSHOT[21]).
- Transfer tissue samples to 0.2 mL tubes
- Add 180 µL of NaOH (50 mM) to each sample
- Heat at 95ºC for 15 min, then allow to cool to room temperature
- Add 20 µL of Tris buffer (1 M, pH 8.0) to neutralize pH.

We include as negative controls ~4 uninjected embryos and HotSHOT components without tissue. We dilute the extract 1:10 by adding 10 µL of the DNA sample to 90 µL of Tris 8.0 (100 mM) prior to PCR.

<u>PCR mix for target amplification</u>

14.1 µl	Water (ultrapure)
2.0 µl	10x Standard Taq Polymerase Buffer (NEB)
0.5 µl	Forward primer (10 µM)
0.5 µl	Reverse primer (10 µM)
0.5 µl	FAM-M13 primer (10 µM)
0.2 µl	dNTP (10 mM)
0.2 µl	Taq polymerase (NEB)
2.0 µL	Embryo template DNA

Amplify samples on a thermocycler: 94°C, 2 min; 94°C, 15 s, 55°C, 15 s, 72°C, 30 s, repeated for 35 cycles; 72°C, 7 min; cool to 4°C. Mix 7 µL of PCR product with 2 µL loading buffer and run on a 2% agarose gel to confirm successful amplification. Gel electrophoresis may occasionally reveal additional bands with sizes that deviate from WT by ~20 bp or more, but most indels are <15 bp in size and cannot be resolved by this method.

Indel mutation detection. To quantify mutation prevalence by size analysis, vortex 20 µL of GeneScan 500 ROX Size Standard (ThermoFisher, 4310361) and add to 1 mL Hi-Di Formamide (ThermoFisher, 4311320). Add 1 µL of each PCR product to a well in a 96-well plate, followed by 9 µL of the ROX-Formamide solution, while empty wells receiving only 10 µL ROX-Formamide to serve as controls. Seal the plate with a rubber septa seal and analyze by capillary electrophoresis on the 3730xl DNA Analyzer (Applied Biosystems) with a default protocol module (GeneMapper50_POP7). We analyze amplicon sizes using Peak Scanner 2 (Thermo Fisher, freely available at resource.thermofisher.com/page/WE28396_2). Peak Scanner enables visualization of PCR amplicon sizes present. Uninjected embryos should have a single peak. Additional peaks in samples from injected embryos are indicative of indels: difference from wildtype size indicates size of insertion or deletion while the height of peaks correlates with its prevalence in the embryo.

To quantify mutation efficiency, we use an online tool, Fragment Analysis (fragmentanalysis.com). We output .csv files from Peak Scanner that contain the size and prevalence of each PCR size variant, which permits the quantification of the fraction of each allele present in each embryo. The website tool is used to establish a "Fragment Analysis Set" and conduct "Standard Fragment Analysis" to access the percentage of DNA fragments that fall into the ranges established by the set. After setting up analysis parameters, the .csv file from Peak Scanner can be uploaded to create a new fragment analysis report. The program quantifies the area under each peak, which is proportional to allele frequency within the mosaic tissue. The ratio of the area under the wildtype peak to sum of areas under all peaks represents the fraction of unmodified alleles. This approach can also be used to identify offspring of injected fish that carry a mutation. When analyzing these animals, it is expected that heterozygotes will display two comparably sized peaks.

PCR products from heterozygotes should be sequenced from each side of the cut site to identify indel location. The sequence of the mutant allele may be inferred from the overlapping sequences: additional chromatogram peaks are found where sequences diverge. The wild-type sequence can be masked and the mutated sequence determined, a process assisted by programs such as Poly Peak Parser[22]. Alternately, TA cloning of PCR products can unambiguously reveal the alternate allele sequence[14]. Animals carrying mutations of likely large effect (e.g., frameshift) should be maintained and bred. Furthermore, insertions or deletions of ≥15 bp can be detected through agarose gel electrophoresis. Using lines carrying such mutations will significantly speed the genotyping process for future generations, as mutant and wildtype amplicons can be readily separated on an agarose gel. If a mutation of small size is recovered, carriers can be detected using PCR primers that bind specifically to either the mutant or wildtype allele. Alternatively, if a restriction enzyme recognition site is created or destroyed by the mutation, these size differences may be detected after PCR and cleavage.

Analysis and Maintenance of Mutant lines

Experimenters may analyze animals at any generation of this process, though caveats apply. As CRISPR/Cas is a robust technology that often leads to high mutation rates, the injected (F_0) fish often exhibit a phenotype[11,14]. However, this founder analysis suffers from potential confounds. First, though CRISPR/Cas is reported to have a low rate of off-target gene modification, it may induce phenotypes due to mutation, particularly at homologous sites in the genome. Second, this approach is also prone to false negatives, as some cells of the mosaic animal carry cells with unaltered DNA, or mutations of weak effect. Third, the variety of mutations created lead to reduced reproducibility. Despite these concerns, we regularly analyze injected animals to obtain preliminary data, refine phenotype tests, and observe unanticipated phenotypes. Further crosses are warranted to obtain animals carrying mutations in all cells. One may intercross F_0 fish, which will result in offspring carrying mutations in all cells, thereby increasing the likelihood of observing a phenotypic effect. Since CRISPR/Cas generates a variety of mutation sizes and sequences even within the germline, each offspring will likely carry a different pair of alleles, complicating interpretations. Furthermore, each parent may contribute mutation(s) in off-target genes to the offspring. Thus, it is important to outcross F_0 animals to wildtypes in order to dilute the effect of off-target effects. Assuming that the on- and off-target loci are unlinked, each generation of outcrossing leads to a reduction by half of the co-inheritance of off-target mutations. Furthermore, unlinked off-target mutations will be inherited by siblings at equivalent rates, providing important controls for effects of this genetic background. Ideally, therefore, crosses of heterozygous F_1 (or later) generation fish will yield control genotypes in addition to homozygous animals for analysis, while controlling for off-target effects. We also recommend analyzing ≥2 independent mutant lines for analysis to ensure that phenotypes are reproducible.

Developing and troubleshooting cichlid gene editing

Obtaining viable injected embryos is a major hurdle to genetic engineering. We have identified several factors that significantly influence the survival rate in microinjection. The width of injection needles is key: bores larger than 12.5 µm are associated with lower post-injection survival rates. This may be due in part to the large puncture created in the embryo and to the increased volume of reagents delivered. In fact, we find that large-bore needles often cause yolk to leak out of embryos, an outcome linked to drastically lower survival. We recommend testing various needle sizes and rigorously screening their sizes when injecting a new fish species. We find that injection of CRISPR reagents into embryos results in a developmental delay of ~12-24 hours, so developmental milestones will not be reached at the same age as in controls (data not shown). We also suggest comparing the survival rates of embryos subjected to varying degrees of manipulation to identify potential causes of low survival. Additional groups may be maneuvered into wells but not injected; punctured by the needle but no reagents delivered; or injected with vehicle. These controls will indicate whether there are issues with embryo handling, injection mechanics, or reagents, respectively. As a first test of CRISPR editing, we recommend injecting gRNA against *Tyrosinase*. This sequence is highly conserved among cichlids, and CRISPR mutation results in an observable, penetrant phenotype of nonpigmented melanocytes. The melanin deficiency remains a stable indicator of mutation in adult mosaic animals. Furthermore, early in development the lost black eumelanin pigment is replaced by a fluorescent molecule(s), presumably from buildup of tyrosine or a derivative[23]. This provides a positive marker of *Tyr1* gene conversion, and permits visual counts of the unmodified and mutant cells. Our results show that CRISPR/Cas9 editing rates at the *Tyr1* locus as determined by tissue biopsy are correlated with the deficiency in melanin. This obvious phenotype can be screened in the embryos as early as 2-3 dpf. This makes the *Tyr1* gRNA-injection a fast and reliable tool to quickly validate CRISPR/Cas activity in vivo, and to troubleshoot problematic reagents and protocols.

Supplementary Material References

1. El-Brolosy, M. A. *et al.* Genetic compensation triggered by mutant mRNA degradation. *Nature* **568**, 193–197 (2019).
2. Ma, Z. *et al.* PTC-bearing mRNA elicits a genetic compensation response via Upf3a and COMPASS components. *Nature* **568**, 259–263 (2019).
3. Zetsche, B. *et al.* Cpf1 Is a Single RNA-Guided Endonuclease of a Class 2 CRISPR-Cas System. *Cell* **163**, 759–771 (2015).
4. Walton, R. T., Christie, K. A., Whittaker, M. N. & Kleinstiver, B. P. Unconstrained genome targeting with near-PAMless engineered CRISPR-Cas9 variants. *Science* **368**, 290–296 (2020).
5. Doench, J. G. *et al.* Rational design of highly active sgRNAs for CRISPR-Cas9-mediated gene inactivation. *Nat. Biotechnol.* **32**, 1262–1267 (2014).
6. Labun, K. *et al.* CHOPCHOP v3: expanding the CRISPR web toolbox beyond genome editing. *Nucleic Acids Res.* (2019) doi:10.1093/nar/gkz365.
7. Alward, B. A. *et al.* Modular genetic control of social status in a cichlid fish. *PNAS* **117**, 28167–28174 (2020).
8. Ata, H. *et al.* Robust activation of microhomology-mediated end joining for precision gene editing applications. *PLOS Genetics* **14**, e1007652 (2018).

9. Mann, C. M. *et al.* The Gene Sculpt Suite: a set of tools for genome editing. *Nucleic Acids Res* **47**, W175–W182 (2019).

10. Varshney, G. K. *et al.* A high-throughput functional genomics workflow based on CRISPR/Cas9-mediated targeted mutagenesis in zebrafish. *Nat Protoc* **11**, 2357–2375 (2016).

11. Hoshijima, K. *et al.* Highly Efficient CRISPR-Cas9-Based Methods for Generating Deletion Mutations and F0 Embryos that Lack Gene Function in Zebrafish. *Developmental Cell* **51**, 645-657.e4 (2019).

12. Varshney, G. K. *et al.* High-throughput gene targeting and phenotyping in zebrafish using CRISPR/Cas9. *Genome research* **25**, 1030–42 (2015).

13. Kidd, M. R. *et al.* Female preference for males depends on reproductive physiology in the African cichlid fish Astatotilapia burtoni. *Gen Comp Endocrinol* **180**, 56–63 (2013).

14. Juntti, S. A. *et al.* A Neural Basis for Control of Cichlid Female Reproductive Behavior by Prostaglandin F2alpha. *Current biology : CB* **26**, 943–9 (2016).

15. Juntti, S. A., Hu, C. K. & Fernald, R. D. Tol2-Mediated Generation of a Transgenic Haplochromine Cichlid, Astatotilapia burtoni. *PLoS One* **8**, e77647 (2013).

16. Jao, L. E., Wente, S. R. & Chen, W. B. Efficient multiplex biallelic zebrafish genome editing using a CRISPR nuclease system. *P Natl Acad Sci USA* **110**, 13904–13909 (2013).

17. Li, M., Dai, S., Liu, X., Xiao, H. & Wang, D. A detailed procedure for CRISPR/Cas9-mediated gene editing in tilapia. *Hydrobiologia* (2020) doi:10.1007/s10750-020-04414-8.

18. Hwang, W. Y. *et al.* Efficient genome editing in zebrafish using a CRISPR-Cas system. *Nat Biotechnol* **31**, 227–229 (2013).

19. Samarut, É., Lissouba, A. & Drapeau, P. A simplified method for identifying early CRISPR-induced indels in zebrafish embryos using High Resolution Melting analysis. *BMC Genomics* **17**, 547 (2016).

20. Brownstein, M. J., Carpten, J. D. & Smith, J. R. Modulation of Non-Templated Nucleotide Addition by Taq DNA Polymerase: Primer Modifications that Facilitate Genotyping. *BioTechniques* **20**, 1004–1010 (1996).

21. Truett, G. E. *et al.* Preparation of PCR-quality mouse genomic DNA with hot sodium hydroxide and tris (HotSHOT). *Biotechniques* **29**, 52, 54 (2000).

22. Hill, J. T. *et al.* Poly peak parser: Method and software for identification of unknown indels using sanger sequencing of polymerase chain reaction products. *Dev. Dyn.* **243**, 1632–1636 (2014).

23. Weber, G. Fluorescence-polarization spectrum and electronic-energy transfer in tyrosine, tryptophan and related compounds. *Biochem J* **75**, 335–345 (1960).
